# Patient satisfaction with e-oral health care in rural and remote settings: a systematic review

**DOI:** 10.1186/s13643-022-02103-2

**Published:** 2022-10-29

**Authors:** Elham Emami, Hermina Harnagea, Richa Shrivastava, Motahareh Ahmadi, Nicolas Giraudeau

**Affiliations:** 1 Faculty of Dental Medicine and Oral Health Sciences, McGill University, 2001 McGill College, suite 500, Montreal, QC Canada; 2 Faculty of Dentistry, Université de Montréal, Montréal, Québec Canada; 3 Present Address: Postgraduate Institute of Medical Education and Research, Chandigarh, India; 4 UMR 5112, CEPEL, CNRS, Université de Montpellier, Montpellier, France

**Keywords:** e-health, e-oral health, Teledentistry, Rural and remote communities, Patient satisfaction

## Abstract

**Background:**

During the past decade e-oral health technology has been used to address the oral health care challenges in rural and remote settings. This review systematically evaluated the literature on patient satisfaction with e-oral health care in rural and remote communities.

**Methods:**

The systematic review included interventional and observational studies published between 1946 and 2021, in the Cochrane Central Register of Controlled Trials, MEDLINE, EMBASE, and Global Health. Patient satisfaction with received oral health care using self-reported measures, at any time after the intervention, was the main outcome of the review. The other primary outcomes were undesirable consequences of the health care (e-health or conventional) such as diagnostic error. The secondary outcomes considered were waiting time, number of visits, travel, and the cost of oral health care. Two independent researchers assessed the risk of bias using the ROBINS-I risk of bias assessment tool for non-randomized studies.

**Results:**

Among 898 studies, 16 studies were included in the review. In most studies reporting patient satisfaction, all patients had shown willingness for teleconsultation for a dental problem and they were mostly satisfied due to saved travel time, saved working days, and prompt treatment onset. Most of the studies acknowledged teledentistry as a cost-effective and cost-saving method. Moreover, the teledentistry consultations showed diagnostic reliability and validity values comparable to conventional dental consultations. The majority of studies were considered level 4 and 3b, due to limited sample populations, analysis based on limited alternatives or costs, non-consistent sensitivity analysis, failure to appropriately control known confounders, and/or failure to carry out an appropriate follow-up of patients.

**Conclusion:**

Available evidence indicates that e-oral health is associated with higher patient satisfaction and has been found to be an effective and reliable method for patients in rural and remote areas. Therefore, in these areas, the use of e-oral health should be encouraged. However, methodological inconsistencies in the current evidence suggest the need for long-term cohort studies and clinical trials, as well as cost analysis on e-oral health in rural settings.

**Systematic review registration:**

The systematic review protocol was registered in the International Prospective Register of Systematic Reviews (PROSPERO) under the registration number CRD42016039942.

**Supplementary Information:**

The online version contains supplementary material available at 10.1186/s13643-022-02103-2.

## Background

Since 2018, the World Health Organization (WHO) has recommended the use of digital technologies in health care sectors under the resolution adopted by the World Health Assembly [1]. The oral health resolution voted by the WHO Executive Board in 2021 includes digital technologies and teledentistry as a tool to translate health policy into action [2]. According to Eysenbach’s definition, “E-Health is an emerging field in the intersection of medical informatics, public health and business, referring to health services and information delivered or enhanced through the internet and other related technologies” [3]. The use of e-health and e-oral health technologies enables effective remote screening, diagnosis, faster referral from primary care to specialist services, reduced amount of travel to urban sites, and increased cost-effectiveness of health care [4–6]. Furthermore, e-oral health has been used as a tool for promoting oral health, preventing dental disease, and improving oral health literacy [7, 8]. It facilitates access to dental care for high-risk populations in underserved communities, such as rural and remote communities, people with special health care needs, and people with low socio-economic status [6, 8]. E-oral health also provides an opportunity to reduce overall cost and improve oral health outcomes [6, 7].

Moreover, e-health and e-oral health technologies also improve patient health care communication and remote education [9, 10]. The effectiveness of e-health has been especially acknowledged during the COVID-19 pandemic [11], and telemedicine and teledentistry have addressed patients’ needs during the closure of dental clinics in this period. Patient-based evaluation of health care is a source of information and a tool for empowerment for health care users [12]. In fact, patients’ satisfaction with various dimensions of health care is a major concern in public and private health care sectors, and has been considered as the “voice of the customer” [13]. Patient satisfaction is a quality care indicator reflecting patients’ experience with received health care services in terms of quality, accessibility, availability, and affordability [14–17]. Evaluation of patient’s satisfaction with health care could also reflect health care disparities especially in the context of rural and remote settings [17]. It has been reported that patients living in rural and remote communities may be dissatisfied with oral health care and face suboptimal oral health care outcomes because of the limited number of dental professionals in these areas, as well as less access to dental care and oral health literacy related to geographical barriers [10, 18, 19]. Furthermore, professional incompetency due to the lack of specialists or peer feedback may be a source of patient dissatisfaction [19–21]. Given that a large number of e-health strategic plans are being developed in rural and remote areas across the world, further investigation on this topic will support policy decision-making and planning for e-oral health programs, which will lead to the improvement of oral health and oral health care in rural and remote areas. However, to our knowledge, no systematic reviews have been carried out on the effect of e-oral health technology on patient satisfaction in rural and remote settings [18, 21–23].

Therefore, this systematic review aimed to answer the following questions:

1. When compared with conventional oral health care, do e-oral health care interventions improve the satisfaction of patients in rural and remote settings with received oral health care?
2. Is the harmful effect of diagnostic errors made in e-oral health care interventions in patients in need of oral health care in rural and remote settings comparable to the effect of such errors in conventional oral health care?
3. To what extent does e-oral health care improve patient satisfaction with care in terms of reducing waiting time, number of visits, travel, and the cost of care for patients in need of oral health care in rural and remote settings, when compared to conventional oral health care?

## Methods

The protocol of this systematic review was registered in PROSPERO (International Prospective Register of Systematic Reviews, registration number CRD42016039942) and was previously published [22]. The PRISMA reporting guidelines were followed [24] (Additional file 1).

### Information sources and search strategy

Electronic literature searches were conducted in summer 2017 and updated in February 2021 in the following databases: Cochrane Central Register of Controlled Trials (The Cochrane Library, current issue), MEDLINE (OVID interface, 1946 onwards), EMBASE (OVID interface, 1974 onwards), and Global Health (OVID interface, 1973 onwards). As described in the published protocol, the search strategy used medical subject headings (MeSH), EMTREEs, and text words related to the field of the study (Additional file 2). It was then peer-reviewed by HH and RS and complemented by hand searching the list of references in the identified publications or relevant reviews. The searches’ procedures were adapted to all databases using the proper syntax, subject headings, and controlled vocabulary considering maximized sensitivity of the search. No language restrictions were used in the search strategies in order to maximize the sensitivity and to identify the number of publications in other languages and verify the existing risk of bias. NICE Evidence and TRIP database were searched for grey literature using subject keywords.

### Inclusion and exclusion criteria

We included English and French language original research studies in the review, with a defined quantitative methodological approach (interventional or observational) including randomized clinical trials, quasi-experimental trials, longitudinal cohorts, and cross-sectional surveys. We excluded case reports, position papers, reviews, and ongoing studies [22].

We adopted the Eysenbach definition of e-health [3]: any type of e-oral health technology that could address the oral health needs of participants in terms of education, consultation, screening, diagnosis, treatment, support, or any other type of application in the field of dental medicine [23], with no limitation in terms of the duration of the intervention and the type of stakeholders involved in the intervention. Conventional oral health care was defined as traditional approaches to oral health care including patients’ education, consultation, disease screening, diagnosis, treatment, and support, or any other type of application in the field of dental medicine [22].

Patient satisfaction with received oral health care using self-reported measures, at any time after the intervention, was the main outcome of the review. The other primary outcome was undesirable consequences of the health care (e-health or conventional) such as diagnostic error. The secondary outcomes considered were waiting time, number of visits, travel, and the cost of oral health care [22].

### Data extraction

The identified articles from search results were transferred to EndNote software. The process of data selection and collection was pilot tested in 10% of randomly selected included articles. Cohen’s kappa test was used to assess the reviewers’ agreement on study eligibility (*k* = 0.878) [22]. Two independent reviewers screened all retrieved titles and abstracts using the inclusion criteria [22] (HH, MA). Discrepancies between reviewers were discussed and resolved through consensus.

The reviewers independently extracted the data from the full text of the included studies by adapting the review form from Effective Practice and Organization of Care (EPOC) Resources for review authors [25], as a data extraction method (Additional file 3).

### Risk of bias in individual studies

Two reviewers (MA, RS) independently assessed the quality of the reports and the risk of bias. For the assessment of experimental studies, the Cochrane Collaboration tool for assessing the risk of bias was used. The assessment of observational studies was performed using the ROBINS-I risk of bias assessment tool for non-randomized studies [26] (Additional file 4). Disagreement was resolved by consultation with a third reviewer (EE).

### Data synthesis

A narrative synthesis was conducted in line with the guidance from the Centre for Reviews and Dissemination [27]. Text and tables summarize and explain the characteristics of the findings in the included studies. The following variables were extracted, and validation checks were performed by HH and RS to assess the accuracy of the extracted fields: (i) lead author and year of publication, (ii) target condition, (iii) study design and sample size, (iv) country and setting of the study, (v) technologies features, (vi) main result, (vii) patient satisfaction measure, and (viii) other outcomes (Table 1). In view of the significant clinical, methodological, and statistical heterogeneity among the studies identified, the data available did not permit meaningful meta-analysis to be performed. According to Haidich, “meta-analysis should be conducted when a group of studies is sufficiently homogeneous in terms of subjects involved, interventions, and outcomes to provide a meaningful summary” [28]. Consequently, we conducted a broad narrative synthesis of the data.

**Table 1:** Characteristics of the included studies and summary of results (*N* = 16)

				**Participants**			**Intervention**		**Outcome**		
**Author/** **year/country**	**Journal**	**Design (assessed by researcher)**	**Field of application**	**Sample size total (gender)**	**Age:** **Mean ± SD,** **(min–max yrs)**	**Source**	**Description of the intervention**	**Type technology**	**Type outcome measure**	**Measurement instrument**	**Results**
1. Patterson and Botchway 1998 [29] Canada	J Can Dent Assoc	Pilot study Cross-sectional (observational)	Dental screening	•137 screened via traditional method •Among them, 32 telehealth screened after 2 months, •27 analyzed as 5 children lost teeth in between the 2 months	N/A	2 Elementary Schools	•Telehealth screenings conducted by dental hygiene students and regional dental officer. Images were transmitted to the Telehealth Centre and screened by the same dental hygienists and dentist who had conducted the initial school screenings. Telehealth screenings were compared to baseline data	LinkCarer System: fully interactive audio and video components via telephone lines	Effectiveness of telehealth technology versus traditional screenings, screening time, screening cost	•Deft/DMFT indices score and number of errors in spreadsheet compilation	•No significant difference between two methods and agreement between these ranged between 89%-100% •Perfect agreement was found for primary teeth to be extracted, permanent missing and permanent filling; very good agreement for primary filled; and moderate agreement for both decay groups
2. Scuffham and Steed 2002 [30] United Kingdom	J of Telemedicine and Tele care	Non-randomized trial for 12 months	Economic evaluation of teledentistry	25	Mean 46 (16–49)	Patients requiring a referral to a specialist in 2 general practices Questionnaires: 14 patients; 18 specialists and 15 GDPs	•Specialist consultation via videoconference compared to specialist outreach visits and patients’ hospital visits at two general dental practices	Videoconference: ISDN connection, Teleconsultation	Costs fixed, direct, indirect	•Questionnaires completed by patients, GDPs, and specialists	•Additional cost for national health service (€36 and €44/patients at both dental practices) compared to outreach visits, cost savings (€270 and €1.54 at both dental practices) compared with hospital visits •Indirect cost savings were higher with teledentistry (79%) and outreach visits (84%) compared to hospital visits •Cost-savings for the NHS, however patients (travel) and general dental practitioners incurred some new costs (time)
3. Ignatius et al 2010 [31] Finland	J of Telemedicine and Tele care	Observational descriptive	Diagnosis and treatment plans	49: 25 professionals: 18 dentists, 2 dental hygienists and 5 nurses; 24 patients	N/A	Patients requiring prosthetic or rehabilitation treatment Central hospital and regional health centers	•Effectiveness of videoconferencing for accurate diagnosis and making treatment plans for rehabilitation treatment	Videoconferencing using standard commercial units via an IP network, at bandwidths of 762 kb/s– 2 Mb/s	Number of diagnosis and treatment planning Satisfaction of dentists and patients	•Dentist examination by videoconference; •Questionnaires	•Videoconferencing equipment functioning was reliable and led to smooth consultations in 24 out of 27 cases •Patients were satisfied. The greater the distance, the higher the satisfaction (*p* < 0.01) •Professionals were also satisfied
4. Herce et al 2011 [32] Spain	J Oral Maxillofac Surg	Evaluative pilot study (multicenter, longitudinal, descriptive)	Evaluation of presurgical management of impacted third molar pathology	97 (52♀ 45♂)	35.07 ± 13	Patients with impacted third molars with no contraindication for extraction under local anesthesia; university hospital and 4 rural dental clinics	•Presurgical management of third molar pathology: dentist examination via PC, information is gathered and sent to Oral and Maxillofacial surgery unit	Store-and-forward telemedicine system (SFTMS)	Clinical effectiveness of SFTMS as a preoperative management and planning tool; waiting intervals; patient satisfaction	•Evaluation of teleconsultations by the maxillofacial surgeon, patient examination and clinical information compared to clinical data registered by PC dentist •Days between visit to PC dentist and day of inclusion on surgical wait list •Patient satisfaction surveys	•The SFTMS is effective tool in the presurgical management of patients •Statistically significant shorter waiting intervals achieved by SFTMS (3.33; 95% CI = 2–4.65 days) in comparison to conventional referral system (28; 95% CI = 24.51–29.6 days) (*p* < .001) •Avoidance of unnecessary hospital visits •No statistical significance in cancellations between telemedicine and traditional system (*p* = 0.76) •Patient satisfaction: 77.3% very satisfied and 22.3% just satisfied
5. Salazar-Fernandez et al 2012 [33] Spain	J Oral Maxillofac Surg	Quasi-experimental Analytical Clinical study	Management of temporomandibular Joint (TMJ) Disorders patients	1052 E: 342 (276♀ 66♂) C: 710 (587♀ 123♂)	E: 38.3 C: 41.08	TMJ Disorders patients in 10 primary care hospitals, Northern Seville	•Using telemedicine system (images and clinical records examined by 2 distant maxillofacial surgeons) as a method for the selection, diagnosis, and treatment of patients with TMJ disorders at remote site compared to conventional hospital consultation	Store-and-forward telemedicine system through the Andalusian Public Health System Intranet (TMJ, ISDR-B, and Frame Relay/ADSL networks) to the e-mail account	1. Clinical effectiveness (rates of diagnosis of myofascial syndrome and/or internal derangement Wilkes Stages I-II-III, internal derangement Wilkes Stages IV–V, other arthropathies; resolved teleconsultations, number of second teleconsultations; rate of referrals to hospitals; mean treatment delay; reduction of number of first hospital visits, 2. Cost (lost working hours/patients) 3. Patient satisfaction	•X-ray images, clinical information, standard •Questionnaires satisfaction surveys	•No statistical differences in clinical effectiveness were found between the two groups (standard vs telemedicine) •Rates of pathologies requiring assistance in the TMJ unit (10.2% vs 11.6%) •Patients requiring non-surgical treatment (89.7% vs 88.4%) •Resolved consultations (88% vs 74.5%; *p* = 0.07) •Second teleconsultation (0.8% vs 4.6%, *p* = 0.07) •Mean cost of lost working hours per patient (16.8 Vs 32.24; *p* < .01) •Mean waiting time was significantly lesser in teleconsultation (2.3 days; 95% CI: 2.2–2.4) compared to standard system 78.6 days; 95% CI: 77.0–80.1) (*p* = .00) •63% patients were very satisfied, 36.7% satisfied, and only 1 patient was unsatisfied
6. Birur et al 2015 [34] India	J of ADA	Descriptive Observational study	Screening	3440 Cohort 1 (targeted screening by health care workers) = 2000 Cohort 2 (Opportunistic screening by dental professionals) = 1440	(> 40)	Adults with high prevalence of oral cancer risk habits Primary health center	•Effectiveness of a mobile-phone based remote oral-cancer surveillance program to detect lesions and capture interpretable images •Dental screening by general dentist and health care workers and specialist diagnosis (reference standard)	Mobile health application for remote oral cancer surveillance (Oncogrid)	Lesion detection, Capture of interpretable images of the oral cavity	•Risk evaluation questionnaire, •Image detection by specialist, •Histological evaluation using WHO classification	•In the targeted cohort showed 45% concordance with specialists and the opportunistic cohort showed 100% concordance
7. Marino et al. 2016 [35] Australia	Journal of Telemedicine and Telecare	Cost-analysis	Screening and caries assessment Cost evaluation	100	N/A	Residential aged care facility in rural areas of the Australian state of Victoria,	•Comparing the cost and benefits of face-to-face patient examination assessments conducted by a dentist with two teledentistry (asynchronous and real time)	Teledentistry	Costs–fixed, direct, indirect	•Costs	•Cost for Teledental asynchronous = AU$32.35/resident (lowest); •Cost for teledental real time = AU$41.28/resident •Cost for face-to-face examination = AU$36.59 /resident
8. Wood et al 2016 (I) [36] USA	J Oral Maxillofac Surg	Cross sectional	Assessing perceived utility and demand for the application of telemedicine for improved patient care	226 GP and 41 OMS	NA	Practicing Virginia Dental Association members on an e-mail list (approximately 2,200) Virginia Society of Oral Maxillofacial Surgery members on an e-mail list (approximately 213)	•Determine the perceived utility and demand for the application of telemedicine	Teledentistry	Perceived utility and demand for the application of telemedicine	•Two distinct questionnaires, one for the non-surgical dental practitioners (GPs) and oral and maxillofacial surgeons (OMS)	•Rural patients had a longer time from referral to OMS consultation (*P* = .003) and traveled longer distances (*P* < .0001); •GPs moderately agreed to the benefits of telemedicine while OMS were mostly neutral •GPs agreed to refer more patients if teleconsultation was used. More referrals would influence OMSs' decision to offer teleconsultations •GPs had neutral opinion on the reliability of teleconsultations, whereas OMS said they would use it if provides equally good consultations as face-to-face consultations
9. Wood et al 2016 (II) [37] USA	J Oral Maxillofac Surg	Retrospective study	Assessing patients for surgical treatment under Anesthesia (triage)	335 (331 ♂ [99%])	Mean 32.5 years (SD = 9.31 years)	Data were collected from a retrospective patient chart review from telemedicine consultations performed between the Virginia Commonwealth University Medical Center and the Virginia Department of Corrections from May 2008 to June 2014	•Efficiency and reliability of telemedicine consultations for preoperative assessment of patients	Data from telemedicine consultations	Chief complaint, history of current illness, and medical history by ‘‘face-to-face’’ video interview, examination by intraoral camera, Radiographic examinations, Physical examination assisted by a nurse or surgical technician at the remote site, estimated cost savings over the 6-year period on comparing with the previous study data at the National Institute of Justice	•Dental electronic health charts	•92.2% of the time practitioners successfully used the data collected for diagnosis and treatment plan •95.9% patients were given an accurate diagnosis and treatment plan •99.6% patients were accurately triaged •98% patients were given adequate medical assessments and underwent surgery as planned •Cost saving was substantial at $134,640 for 6 years
10. Petruzzi and De Benedittis 2016 [38] Italy	Oral Surg Oral Med Oral Pathol Oral Radiol	Cross-sectional Observational study	Diagnosis	96 65 rural	N/A	339 clinical images relating to the 96 cases sent by eighty clinicians (general dental practitioners, hygienists, and physicians) and patients, or their relatives 92/96 patients (96%) attended Oral Medicine unit for a clinical examination In 45 cases a biopsy was performed	•Clinical images were spontaneously sent to the authors’ smartphones via WhatsApp. Images were reviewed by two oral medicine experts. The patients then underwent an oral mucosa examination at the clinic, where all biopsied lesions were examined histopathologically by an independent pathologist	WhatsApp messenger mobile application version 2.10 or higher. One examiner used an iPhone 4 s (3.5″ screen) and the other a Galaxy S III (4.8″ screen)	Agreement rates between TM oral medicine expert’s diagnosis and clinicopath assessment	•Percentage calculation	•316 photos (93%) were good quality photos •Telemedicine impressions by the two oral medicine experts agreed with the clinicopathologic assessment for 82% cases, with an inter-rater reliability of 100% •Agreement rate for traumatic cases was 95%, for infectious lesion 96%, for preneoplastic/neoplastic disorders 71%, for autoimmune 82% and for non-pathological conditions was 67%,
11. Purohit BM et al. 2016 [39] India	Journal of Public Health Dentistry	Cross-sectional study	Screening/ dental caries assessment	139 (62♂, 77♀	12 years	School children from same racial/ethnic group located in the region of the outreach health centers of Bhopal district	•Standardized video recording of the oral cavity for caries assessment	A Sony Xperia smart phone with an 8-megapixel camera, 720 * 1,280-pixel resolution, and LED flashlight was used to standardise video recording of the oral cavity. The video files were stored in MP4 format, with a duration of 40 s and a file size of 60 MB, respectively	Mean DMFT	•Visual tactile and video-graphic assessments	•Mean DMFT was 2.47 ± 2.01 by visual tactile and 2.46 ± 1.91 by video-graphic assessment (*p* = 0.76) •Fair agreement between visual tactile and video-graphic assessment (intraclass correlation coefficient = 0.56) •Video-graphic assessment: Sensitivity and specificity values were 0.86 and 0.58 respectively. Positive and negative predictive values were 0.90 and 0.48 respectively (area under the curve = 0.69)
12. Estai M et al. 2017 [40] Australia	Australian Health Review	A cost-minimization analysis	Cost analysis Screening	2.7 million children	5–14 years	Australian school children (Australian Bureau of Statistics)	•Compare the costs of teledentistry and traditional dental screening approaches	Teledentistry	The fixed costs and the variable costs, including staff salary, travel and accommodation costs, and cost of supply Direct and indirect costs	•Costs	•Total estimated cost of teledentistry model = $50 million •The fixed cost of teledentistry = $1 million and fixed cost of staff salaries = $49 million •Total annual saving with the teledentistry was $85 million. Estimated staff salary saved = $56 million, and the estimated travel allowance and supply expenses avoided = $16 million and $14 million respectively •Teledentistry cost an average of $19 per child, compared to $41–187 per child for traditional screening, depending on the distance from residence
13. Teoh J et al. 2018 [41] Australia	Telemedicine and e-health	A model-based analysis was conducted to determine the potential costs of implementing teledentistry at the hospital	Assess the use of Teledentistry	367 Teledentistry appropriate consultations	NA	Royal Children’s Hospital for rural and regional patients	•Assess the use of Teledentistry in delivering specialist dental services •Conduct an economic evaluation by building a decision model to estimate •Costs and effectiveness of Teledental consultations compared with standard consultations at the hospital	Teledentistry	Timely consultations (whether the patient presented within an appropriate time according to the recommended schedule)	•Review of dental records of orthodontic or paediatric dental consultations at the hospital •Cost-effectiveness analysis comparing teledentistry with the traditional method of consultation	•241(65.7%) consultations were timely •The cost saving with teledentistry consultation was A$136.95 per appointment more compared to hospital consultations •With teledentistry, there will be a societal cost saving of $3,160.81 for every timely cleft lip and palate consultation and hospital consultations could have freed up 36.7 days of clinic time
14. Tynan et al. 2018 (I) [42] Australia	BMC Health services research	A mixed method comparison study	Impact and experience of an integrated oral health program utilising tele-dentistry and Oral Health Therapists (OHT)	27 residents (10♂, 17♀) One focus group (5) + 8 Interviews (gender non-specified)	Mean 77.09 (34–101) 82.4 (44–97)	A total of 252 audits were complete across nine residential aged care facilities (111 audits at facilities with integrated oral health and 141 audits at facilities without integrated oral health Out of the 27 participants, 7 were from residential aged care facilities with integrated oral health program and 20 were from facilities without program Participants for qualitative data included 5 nurses from facilities with integrated program and 8 nurses from facilities without the program	•Comparison between facilities with and without an integrated program by Audits, GOHAI surveys, 1 FG + 8 interviews	Tele-dentistry (a dentist for a remote real-time oral examination if required) The OHT, specifically trained in manipulating the intraoral camera, can simultaneously communicate with a remotely located dentist	Comparison facilities with and without integrated oral health program	•Audit •Qualitative content analysis •GOHAI questionnaires	•Audit comparison of facilities with integrated oral health program implemented and with integrated programs showed better compliance with oral health standards at integrated facilities. (More satisfactory oral health plans, 89.2% vs 75.2%; *p* = 0.005) •Mean GOHAI score 50.6 ± 5.1 vs 51 ± 5 indicating poor oral health quality of life •Thematic analysis showed improvements in importance placed on oral health, better access to services and training, and decreased disruption in facilities with integrated programs
15. Tynan et al. 2018 (II) [43] Australia	Aust. J. Rural Health	A quality improvement study incorporating pre- and post chart audits and pre- and post consultation with key stakeholders, including staff and residents, expert opinion on cost estimates and field notes were used	Screening via the oral health therapist and teledentistry appointment	116	N/A	One regional and three rural residential aged care facilities situated in a non-metropolitan hospital and health service in Queensland	•Audits comparison between facilities with and without an integrated program •Perceived impacts on staff and residents •Cost comparison	Teledentistry	Comparison facilities with and without integrated oral health program	•Number of appointments avoided at an oral health facility •Feedback on program experience by staff and residents •Compliance with oral health care plan implementation •Observations of costs involved to deliver new service	•Increase up to 96% of residents with an appropriate oral health care plan was observed •Positive feedback was received from staff, residents, and their families •Staff and managers reported increased awareness of residents’ oral health needs and prevention requirements; improved access to resources for oral health management; and savings from reducing the need to transport residents to an oral health facility •Reduced disruption to high care residents; •Positive cultural change in staff toward oral health care •Initial screenings by the therapist resulted in potential problems being identified and addressed earlier than in the past
16. Vinayagamoorthy et al. 2019 [44] India	Aust. J. Rural Health	An observational cross‐sectional study	Preventive screening of oral potentially malignant Disorders	131 64.1%♀ + 35.9%♂	Mean (SD) age of 37.34 years (11.31)	Primary care setting in Udupi District, Karnataka, South India	•Clinical oral examination followed by photo capture of five areas of the patients' mouth	A Samsung Note 2 mobile phone with an 8‐megapixel camera and a display with a resolution of 720 × 1280 pixels with autofocus (Samsung, Seoul, South Korea) was used The photo messaging feature of the WhatsApp messenger (Version 2.17.190; WhatsApp, Mountain View, CA, USA) Both the examiners examined the images in their mobile phones (Android smartphones with a display size of 5.50″ and a display resolution of 1080 × 1920 pixels) separately with the display brightness at 50%	Reliability measures for the use of a photo messaging service in diagnosing oral potentially malignant disorders, as compared to the clinical examination	•Reliability of an exact diagnostic match of lesions between the examinations •Inter‐examiner and intra‐examiner reliability of the clinical oral examination and photographic examination •Accuracy, sensitivity, specificity, positive predictive value, and negative predictive value •Agreement between the two examiners for the diagnosis based on photo messaging	•When lesions were classified as normal or abnormal, the reliability between diagnoses for Examiners 1 and 2 based on photo messaging and clinical oral examination was 0.68 and 0.67, respectively •Sensitivity for examiners 1 and 2 were 98.5% and 99.04%, respectively, and specificity was 72% and 64%, respectively •When the agreement between photo messaging and clinical oral examination for an exact diagnostic match was evaluated, the reliability was 0.59 and 0.55 for Examiners 1 and 2, respectively •Sensitivity for Examiners 1 and 2 were 98.1% and 98.7%, respectively, and specificity was 64% and 52% respectively

## Results

### Study selection

**Fig. 1:**
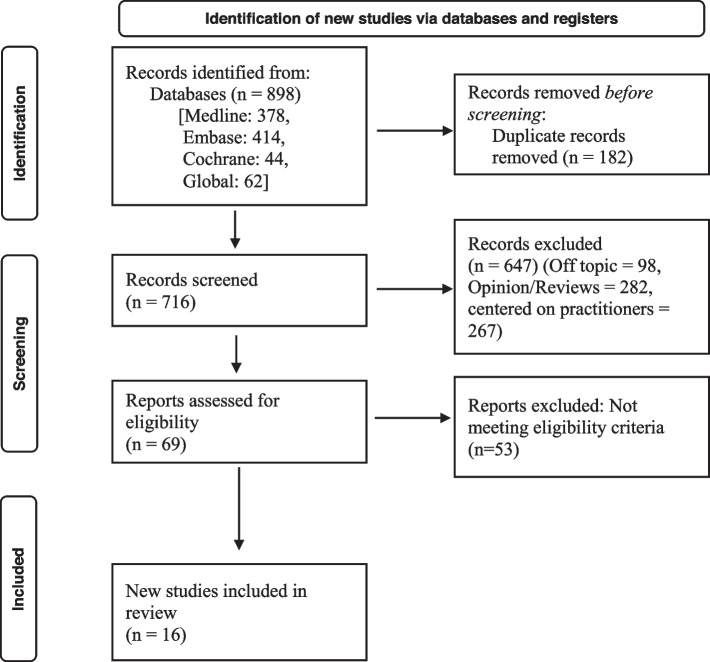
PRISMA flow diagram (Adapted from PRISMA 2020 statement)

The electronic search yielded a total of 898 studies from MEDLINE (*n* = 378), EMBASE (*n* = 414), COCHRANE (*n* = 44), and GLOBAL (*n* = 62). After removing duplicates, 716 studies remained for screening. The screening based on title and abstract resulted in the exclusion of 647 articles, with the main reasons for elimination being that the studies were not related to dentistry and/or did not have a quantitative design. Of the 69 potentially eligible studies, full-text screening led to a further exclusion of 53 studies, which were deemed not relevant to the study aim (Fig. 1). The excluded studies were not centered on patients but rather, they were conducted only among dental professionals and did not consider rural and remote aspects. A total of 16 studies were included in this review.

### Study characteristics

The majority of the studies were conducted between 2010 and 2019 [31–44], with only two prior studies carried out in 1998 and 2002 [29, 30]. In total, five studies were from Australia [35, 40–43], three from India [34, 39, 44], two studies were conducted in the USA [36, 37], two in Spain [32, 33], one in Canada [29], one in the UK [30], one in Italy [38], and one in Finland [31]. The types of studies comprised non-randomized clinical trials [30, 33], observational studies [31, 34, 36, 37, 39, 42–44], pilot intervention studies [29, 32, 38], and cost analysis [35, 40, 41]. Most studies used teledentistry consultations, either live or *store and forward* [29, 30, 32, 33, 35, 37, 40–43], and other studies used smartphone-based applications like *WhatsApp* [38, 44], the *Oncogrid* application for oral cancer surveillance [34], and videographic examination [39].

In several studies, teledentistry-based general dental examination and screening were done using intraoral cameras [29–31, 39, 42, 43]. In other studies, specialized dental services were provided such as oral and maxillofacial surgery for impacted third molar pathology, cleft lip and palate patient management, temporomandibular joint disorders [32, 33, 36, 37, 41], oral pathology for preventive screening of oral potentially malignant disorders [44], oral medicine and diagnosis for oral cancer surveillance [34, 38], and prosthodontics for dental prosthetics and oral rehabilitation [31]. All of these specialized dental services primarily used the teledentistry model for specialist teleconsultations, disease screening, diagnosis and surveillance, treatment planning, preoperative assessment and management of patients requiring operative procedures, as well as referrals [31–34, 36–38, 41, 44].

### Synthesis of the results

#### Patient satisfaction

In three studies reporting patient satisfaction, 63 to 78% of patients were very satisfied with e-oral health care and 22 to 37% were satisfied [31–33]. Only one study reported dissatisfaction in one patient (0.3%) [33]. All patients in these studies had shown willingness for teleconsultation for a dental problem [31–33]. As per Herce et al., 77.7% patients out of a total of 90 patients were very satisfied with this type of care, 22.3% patients were just satisfied, and no patient was unsatisfied [32]. Similarly, Salazar-Fernandez et al. [33] reported that 63% of 283 patients were very satisfied, 36.7% were satisfied, and only 1 patient was unsatisfied. Patients were satisfied with such consultations due to saved travel time, saved working days, and prompt treatment onset [33]. In addition, in the study by Ignatius et al. [31], 65% of 24 patients rated their satisfaction with the teleconsultation as 9 or 10 on a scale of 4 (worst) to 10 (best).

These studies reported association of patient satisfaction with travel time [31], prompt treatment initiation, and workdays not lost [33]. As per Ignatius et al. [31], patient satisfaction was associated with the travel distance: the greater the distance of the patient's residence from the hospital, the greater the patient's satisfaction with e-oral health.

Moreover, Ignatius et al. reported that dental professionals were satisfied with the performance of teledentistry devices [31]. Furthermore, in a study conducted by Wood et al. [36], general dental practitioners moderately agreed with benefits of teledentistry and expressed a desire to refer more patients through telemedicine consultations. While oral and maxillofacial surgeons were mostly neutral, they acknowledged that more referrals would influence their decision to provide telemedicine consultations and implement teledentistry in their practice [36].

#### Harmful effect of diagnostic errors made in e-oral health care interventions

There were no studies that found harmful effects, but five studies found that the e-oral health technology was reliable and in acceptable agreement with the standard consultations. According to Wood et al., 92.2% of the time, the practitioners were successful in making the diagnosis and treatment plan by using the teleconsultation data [37]. Nearly 96% of the patients were given an accurate diagnosis and treatment plan, 99.6% of patients were triaged correctly, and 98.0% were given sufficient medical and physical assessment and immediately underwent surgery after teleconsultation [37].

Petruzzi et al., Patterson et al., Vinayagamoorthy et al., and Purohit BM et al. reported significant agreement between teledentistry consultation and clinicopathologic examination [29, 38, 39, 44]. According to Vinayagamoorthy et al., substantial agreement was found when the lesions were dichotomized as normal and abnormal (examiner 1 and 2, K reliability: 0.68 and 0.67, sensitivity: 98.5% and 99.04%, specificity: 72% and 64%), but slightly reduced when assessed for the exact diagnostic match (examiner 1 and 2, K reliability: 0.59 and 0.55, sensitivity: 98.1% and 98.7%, specificity: 64% and 52%) [44]. Birur et al. reported 45% and 100% concordance with the specialists in the targeted cohort and opportunistic cohort respectively [34]. In a study by Purohit BM et al., the sensitivity and specificity were 0.86 and 0.58 for videography-based teledentistry assessment [39].

#### Impact on waiting time, number of visits, travel, and the cost of care for patients

According to Herce et al. [32], the mean waiting interval for patients managed through teledentistry was 3.33 days since the visit to the primary care dentist compared to 28 days for those managed through the conventional referral system [32]. The cancellation rate on-the-day of surgery for telemedicine was 7.8% and for the conventional system was 8.85% [32]. Salazar-Fernandez et al. [33] found that both the teleconsultations and hospital visits were clinically effective [33]. No statistically significant difference between the two techniques was noted for the rates of pathologies requiring assistance, patients requiring nonsurgical treatment, resolved consultations, and second teleconsultations [33]. The effectiveness and efficiency also varied with the distance of the patient’s residence from the hospital [37, 41]. Teoh et al. [41] performed analysis of subgroups determined a priori, based on the distance travelled by the study participants (< 50 km, 50–80 km, 81–150 km, 151–225 km, and > 225 km). Based on the cost-effectiveness analysis, these authors concluded that the greater the distance between the patient's residence and the hospital, the more cost-effective and time-saving teledentistry intervention would be [41]. For instance, the cost difference between tele- and hospital consultations at > 225 km distance was AU$ 458.85, the incremental cost-effectiveness ratio was − 10,550.45, the hours saved were 5.02, and the total distance saved was 450.03 km [41]. Furthermore, this study showed that patients could save an average of 2 h and 21 min of travel time and 178.6 km of travel distance [41]. However, this treatment modality was not as cost effective if a patient resided within 50 km of the hospital [41]. As per the study by Wood et al. [37], average estimated distance of the patients from the clinic was 50 miles and the average roundtrip cost estimate of driving this distance was approximately $60.00 per patient.

As per Ignatius et al. [31] and Scuffman and Steed [30], the additional training of the general dental practitioners and familiarity with equipment and procedures were associated with better teledentistry related outcomes. Training may have higher initial cost but it can be cost-effective in the long run [30]. Birur et al. also reported better concordance in the presence of trained onsite health workers such as in the diagnosis and surveillance of oral cancer [34].

Seven studies acknowledged teledentistry as a cost-effective and cost-saving method. A study by Estai et al. [40] compared traditional dental screening at school with teledentistry using a cost-minimization analysis. This study demonstrated the ability of teledentistry in minimizing the cost; for instance, the total estimated cost and fixed cost of the teledentistry model was $50 million and the estimated annual reduction with the teledentistry model was $85 million, which included staff salary savings, travel allowance avoided, and supply expenses avoided [40]. Similarly, cost analysis by Marino et al. [35] showed that asynchronous teleconsultation was the lowest cost service model with AU$32.35 cost per resident compared to traditional face-to-face (average cost: AU$36.59 per resident) and real-time (average cost: AU$41.28 per resident) consultations.

According to Wood et al., consultation by telemedicine for 255 patients and eliminating in-office consultation saved a significant amount equivalent to $134,640 [37]. In a model-based analysis, in Teoh et al. [41] the expected cost per consultation for conventional care was AU$431.29 and that for teledentistry was AU$294.35, saving an average AU$136.94 in societal costs per consultation. They also mentioned that teledentistry would save AU$50,258.92 in total costs per year, and that costs to the patient were reduced by 69% [41]. Comparing the conventional care in hospital to teledentistry, the largest difference in cost savings was the costs to the patient including their transportation, accommodation, and lost productivity costs, amounting to AU$70,719.19 [41]. The sensitivity analysis after adjustment of potential variables showed that teledentistry is a cost-saving option to society with the saving of $3,160.81 for every timely cleft lip and palate consultation compared to hospital consultation [41]. Salazar-Fernandez et al. [33] reported the mean cost of lost working hours per patient was significantly less (50%) in teleconsultation (16.8 h) compared to the standard system (32.24 h).

Tynan et al. [43], on the other hand, compared three cost scenarios: screening by an oral health therapist in a residential aged care facility, teledentistry in a residential aged care facility, and resident attendance at an oral health clinic. Screening by an oral health therapist was deemed the lowest cost scenario when compared to the other two since the teledentistry setup in scenario 2 and dentist time in scenario 3 increased total costs [43]. In a cost-minimization analysis by Scuffham and Steed [30], the cost of teledentistry was compared with two modes, outreach visits (specialist regularly visiting the two remote communities) and hospital visits. In both the communities, teleconsultation was associated with additional costs to the health organization and society compared with outreach visits [30]. However, the cost savings for teleconsultation varied between the communities compared to hospital visits. In one community, cost savings were higher due to more travel time and travel cost [30]. Nonetheless, the researchers concluded that teledentistry would be a cost-effective tool for the health organization in the long run [30].

#### Risk of bias assessment

Additional file 4 presents the risk of bias assessment tool in individual studies. Thirteen of the selected studies were found to have moderate risk of bias, and two other studies had critical risk in the overall assessment [29–35, 37–44]. One article was found to be ineligible for performing risk for bias assessment using the ROBINS-I tool [36]. Table 2 shows the level of evidence according to the Oxford Centre for Evidence-Based Medicine [45]. The majority of studies (11 out of 16) were considered level 4 and 3b.

**Table 2:** Selected articles’ levels of evidence

Author/year/country/	Level of evidence (Oxford Centre for EBM 2009)
1. Patterson and Botchway/1998/Canada [29]	4
2. Scuffham and Steed/2002/United Kingdom [30]	3b
3. Ignatius et al./2010/Finland [31]	3b
4. Herce et al./2011/Spain [32]	1b
5. Salazar-Fernandez et al./2012/Spain [33]	1b
6. Birur et al./2015/India [34]	1b
7. Marino et al./2016/Australia [35]	2b
8. Wood et al./2016 (I)/USA [36]	4
9. Wood et al./2016 (II)/USA [37]	2b
10. Petruzzi and De Benedittis./2016/Italy [38]	4
11. Purohit BM et al./2016/India [39]	4
12. Estai M et al./2017/Australia [40]	3b
13. Teoh J et al./2018/Australia [41]	3b
14. Tynan et al./2018 (I)/Australia [42]	4
15. Tynan et al./2018 (II)/Australia [43]	4
16. Vinayagamoorthy et al./2019/India [44]	4

[Ref: Centre for Evidence-Based Medicine. Levels of Evidence. Oxford: Oxford Centre for Evidence-Based Medicine; 2009. https://www.cebm.ox.ac.uk/resources/levels-of-evidence/oxford-centre-for-evidence-based-medicine-levels-of-evidence-march-2009. Accessed 20 October 2020]

## Discussion

Patients’ satisfaction with access to care in rural and remotes areas is one of the most important objectives of digital health. The findings of this systematic review suggest that 63% to 78% of patients living in rural and remote areas were very satisfied with e-oral health care interventions [31–33]. This satisfaction was mainly attributed to clinical effectiveness in making the diagnosis and treatment plan, less waiting time, prompt treatment onset, cost effectiveness, saved travel time, and saved working days [32, 33, 35, 37, 40–43]. Moreover, studies reporting on agreement of teledentistry consultation versus conventional dental consultations found comparable diagnostic reliability and validity values [32, 33, 35, 41].

Previous research has reported a rural–urban disparity regarding patients’ satisfaction with oral health care [46, 47]. Additionally, literature suggests that rural residents could have a higher level of satisfaction with oral health care services if they receive these services in a timely manner in their vicinity. For instance, a study on patient satisfaction with emergency oral health care services in rural Tanzania reported satisfaction among 92.7% of patients because of availability of these services in their area [48]. Another study reported 80% satisfaction among patients visiting a rural dental institution in India [49].

The included studies also measured a wide range of other outcomes that were positively associated with e-oral health that we are not reporting here since they were not included in the published protocol. These outcomes include dental professionals’ satisfaction and perceptions about teledentistry [31, 36], perceived utility and demand for the application of telemedicine [36], surgery cancellation rate [32], number of appointments avoided at an oral health facility [43], and compliance with oral health care plan implementation [43].

The available emerging evidence and recent systematic reviews on e-oral health suggest that this technology is effective in promoting oral health and preventing dental problems, and that it has acceptable diagnostic performance [50–52]. Some authors have reported that tele-disease screening, tele-diagnosis, tele-consultations, treatment planning, and referrals, are comparable to face-to-face dentist-patient interaction [53, 54]. Additionally, according to previous studies these technologies seem to be beneficial in school-based programs, long-term care facilities, and areas with limited access to care, including rural and remote areas. However, in the absence of real comparative data it is hard to know how relevant these results are.

E-health services in rural and remote areas have been used for mental health, oncology, geriatrics, paediatric care, trauma treatment, specialist rheumatologist services, wound care management, and chronic disease management [55, 56]. Similar to our results, these e-health services were also found to be effective in rural areas in terms of better patient’s and professional’s satisfaction and acceptance, access to care, convenience, reliability, and reduced overall cost and travel time [55–57]. However, the success of e-health services in rural areas also depends on design and implementation of these services, information and technological support, as well as user training of the health personnel [57].

This systematic review reveals that multiple digital tools such as *WhatsApp*, emails, and videoconferencing have been used to connect oral health professionals and patients. This shows the necessity to work on formal teledentistry platforms. Furthermore, the type of digital tools will influence the quality of the diagnosis and the treatment plan. On the other hand, patients’ knowledge concerning the use of digital technologies is an important aspect and may influence patients’ expectation and satisfaction with the use of such technologies.

The satisfaction of patients may also be influenced by the type of dental care. For example, the use of teledentistry for early detection of oral cancer may result in higher patient satisfaction when compared with the tele-monitoring of the outcomes of orthodontic care. In fact, the use of digital technology should be encouraged in the prevention of oral diseases and improving access to care. According to the French national law on telemedicine enacted in 2009, the telemedicine activities should take into account “the deficiencies in the provision of care due to insularity and geographical isolation” [58]. The WHO *mOralHealth* program [59] is an example of strategies that encourage worldwide e-oral health policies and programs to improve oral health literacy and access to care.

The recent development of e-health technologies and their integration and implementation in primary oral health care by interdisciplinary teams has the potential to address the dental needs of individuals in remote and rural communities, to provide satisfaction to these individuals, and to alleviate the burden of access to care. The findings also have the potential to empower the isolated dental workforce working in rural and remote zones across the world.

However, this review is limited due to its narrow inclusion criteria in regard to language and inclusion of various study designs, and caution should be taken when interpreting the results. Moreover, based on the level of evidence (Oxford Centre for EBM) and ROBINS-I assessments, most of the included studies had moderate or low quality as well as moderate to critical risk of bias. Furthermore, the reporting of outcomes in the included studies varies considerably. Although ROBINS-I is a comprehensive and rigorous tool for assessing bias risk, its reliability is questioned due to a lack of agreement among the examiners [60]. These findings may affect the overall conclusions drawn in this study.

Our findings indicate significant inconsistencies in the methodologies of the included studies in terms of study setting, study design, sampling, data collection, and data analysis. Therefore, future high-quality studies using a mixed-method research design are required to provide quality data especially from end users’ perspectives. Quantitative data of the mixed methods should include randomized controlled trials with valid instruments allowing scoring patient satisfaction whereas the qualitative element can aid in gaining in-depth understanding of patients’ satisfaction with e-oral health. Standardized economic analyses are also required. Furthermore, future research on e-oral health should consider patient-centered oral health care, patient experiences with care, and the cost effectiveness of these technologies, particularly in underserved areas. Further research is needed to understand the role of e-oral health technologies in addressing rural oral health disparities. Moreover, research into the application of these technologies in academic settings will shed light on their significance in education.

## Conclusion

Our results suggest that patient satisfaction could be associated with several modalities of e-oral health care. E-oral health seems a feasible option for providers who want to contribute to oral care services in rural and remote areas. However, only speculative conclusions can be drawn based on the quality of the included studies, implying that long-term robust cohort studies and clinical trials, as well as cost assessments on e-oral health in rural settings, are required in the future. As telehealth continues to be developed, special care should be given to incorporate features that further enable patients’ satisfaction and acceptance. On the other hand, as more patients are using telehealth, additional training for dentists is an important part of assuring better positive outcomes for patients.

## Supplementary Information

**Additional file 1.** PRISMA 2009 Checklist.

**Additional file 2.** Appendix 2. Search methodology.

**Additional file 3.** Effective Practice and Organisation of Care (EPOC). Data collection form. EPOC Resources for review authors. Oslo: Norwegian Knowledge Centre for the Health Services; 2013.

**Additional file 4.** The Risk Of Bias In Non-randomized Studies–of Interventions (ROBINS-I) assessment tool.

## Data Availability

Not applicable.
