# Supplementary material for: Patient satisfaction with e-oral health care in rural and remote settings: a systematic review
Source: Syst Rev. 2022 Oct 29;11:234. doi: 10.1186/s13643-022-02103-2 (PMC9617039; doi:10.1186/s13643-022-02103-2)
Supplement: Supplementary file 2 — Additional file 2. Appendix 2. Search methodology. [file 13643_2022_2103_MOESM2_ESM.docx]

| **#** | 1. **MEDLINE – 2. Embase - 3. Cochrane – 4. Global Searches / Combined searches: 5 = 1 AND 3; 6 = 2 AND 3 AND 4; 7 = 5 OR 6** |
| --- | --- |
| 1.1 | (mobile dentistry or teledentistry or tele-dentistry or edentistry or mdentistry or e-dentistry or m-dentistry or remote dental or dental telecommunication?).mp. |
| 1.2 | exp telemedicine/ or exp remote consultation/ or exp Videoconferencing/ or (mobile health or telehealth or telemedicine or tele-health or tele-medicine or ehealth or mhealth or e-health or m-health or teleconsult* or telepatholog* or telemonitor* or teleconferenc* or videoconferenc* or medical telecommunication? or m-patient* or mpatient* or remote consult* or tele-consult* or tele-patholog* or tele-monitor* or tele-conferenc* or video-conferenc* or medical tele-communication? or remote consultation or telediagnos* or tele-diagnos* or teleradiolog* or tele-radiolog* or teleradiograph* or tele-radiograph* or teleradiotherap* or tele-radiotherap* or telesurg* or tele-surg* or teletherap* or tele-therap*).mp. |
| 1.3 | Rural Health/ or exp Rural Health Services/ or Rural Population/ or Hospitals, Rural/ or (rural or remote or country area or country areas or countryside? or country-side? or aboriginal or native? or tribe? or tribal or indigenous or northwest territor* or nunavut or yukon territory or nunavik or first nation?).mp. |
| 1.4 | exp dentistry/ or exp oral health/ or exp mouth diseases/ or exp Dental Care/ or exp dental caries/ or exp education, dental/ or exp diagnosis, oral/ or (dental or dentist* or endodonti* or orthodont* or periodont* or prosthodont* or odontolog* or pedodont* or paedodont* or oral or tooth or teeth or mouth).mp. |
| 2.1 | (mobile dentistry or teledentistry or tele-dentistry or edentistry or mdentistry or e-dentistry or m-dentistry or remote dental or dental telecommunication?).mp.. |
| 2.2 | exp telehealth/ or exp videoconferencing/ or exp teleconference/ or (mobile health or telehealth or telemedicine or tele-health or tele-medicine or ehealth or mhealth or e-health or m-health or teleconsult* or telepatholog* or telemonitor* or teleconferenc* or videoconferenc* or medical telecommunication? or m-patient* or mpatient* or remote consult* or tele-consult* or tele-patholog* or tele-monitor* or tele-conferenc* or video-conferenc* or medical tele-communication? or remote consultation or telediagnos* or tele-diagnos* or teleradiolog* or tele-radiolog* or teleradiograph* or tele-radiograph* or teleradiotherap* or tele-radiotherap* or telesurg* or tele-surg* or teletherap* or tele-therap*).mp. |
| 2.3 | exp rural health care/ or rural population/ or rural area/ or exp indigenous people/ or (rural or remote or country area or country areas or countryside? or country-side? or aboriginal or native? or tribe? or tribal or indigenous or northwest territor* or nunavut or yukon territory or nunavik or first nation?).mp. |
| 2.4 | exp dentistry/ or exp dental procedure/ or exp mouth disease/ or (dental or dentist* or endodonti* or orthodont* or periodont* or prosthodont* or odontolog* or pedodont* or paedodont* or oral or tooth or teeth or mouth).mp. |
| 3.1 | (mobile dentistry or teledentistry or tele-dentistry or edentistry or mdentistry or e-dentistry or m-dentistry or remote dental or dental telecommunication?).mp. |
| 3.2 | (mobile health or telehealth or telemedicine or tele-health or tele-medicine or ehealth or mhealth or e-health or m-health or teleconsult* or telepatholog* or telemonitor* or teleconferenc* or videoconferenc* or medical telecommunication? or m-patient* or mpatient* or remote consult* or tele-consult* or tele-patholog* or tele-monitor* or tele-conferenc* or video-conferenc* or medical tele-communication? or remote consultation or telediagnos* or tele-diagnos* or teleradiolog* or tele-radiolog* or teleradiograph* or tele-radiograph* or teleradiotherap* or tele-radiotherap* or telesurg* or tele-surg* or teletherap* or tele-therap*).mp. |
| 3.3 | (rural or remote or country area or country areas or countryside? or country-side? or aboriginal or native? or tribe? or tribal or indigenous or northwest territor* or nunavut or yukon territory or nunavik or first nation?).mp. |
| 3.4 | exp dentistry/ or exp oral health/ or exp mouth diseases/ or exp dental caries/ or exp diagnosis, oral/ or (dental or dentist* or endodonti* or orthodont* or periodont* or prosthodont* or odontolog* or pedodont* or paedodont* or oral or tooth or teeth or mouth).mp. |
| 3.1 | (mobile dentistry or teledentistry or tele-dentistry or edentistry or mdentistry or e-dentistry or m-dentistry or remote dental or dental telecommunication?).mp. |
| 3.2 | (mobile health or telehealth or telemedicine or tele-health or tele-medicine or ehealth or mhealth or e-health or m-health or teleconsult* or telepatholog* or telemonitor* or teleconferenc* or videoconferenc* or medical telecommunication? or m-patient* or mpatient* or remote consult* or tele-consult* or tele-patholog* or tele-monitor* or tele-conferenc* or video-conferenc* or medical tele-communication? or remote consultation or telediagnos* or tele-diagnos* or teleradiolog* or tele-radiolog* or teleradiograph* or tele-radiograph* or teleradiotherap* or tele-radiotherap* or telesurg* or tele-surg* or teletherap* or tele-therap*).mp. |
| 3.3 | (rural or remote or country area or country areas or countryside? or country-side? or aboriginal or native? or tribe? or tribal or indigenous or northwest territor* or nunavut or yukon territory or nunavik or first nation?).mp. |
| 3.4 | exp dentistry/ or exp oral health/ or exp mouth diseases/ or exp dental caries/ or exp diagnosis, oral/ or (dental or dentist* or endodonti* or orthodont* or periodont* or prosthodont* or odontolog* or pedodont* or paedodont* or oral or tooth or teeth or mouth).mp. |

Appendix 2 : Search methodology
